# Understanding inner self-states in eating disorders: preliminary findings from a structured elicitation study

**DOI:** 10.1007/s40519-026-01857-1

**Published:** 2026-04-15

**Authors:** Paolo Meneguzzo, Natalia Seijo, Anna Pillan, Enrica Bucci, Alice Garolla, Anna Marzotto, Francesca Buscaglia, Patrizia Todisco

**Affiliations:** 1 Department of Neuroscience, University of Padova, Via Giustiniani 2, 35128 Padua, Italy; 2 Padova Neuroscience Center, University of Padova, Padua, Italy; 3 NS Center Psychotherapy and Trauma, Ferrol, Spain; 4 Eating Disorder Unit, Casa di Cura “Villa Margherita” – KOS Group, Arcugnano, Vicenza, Italy; 5 Psycho-Nutritional Center, Vicenza, Verona Italy

**Keywords:** Eating disorders, Self-parts, Metaphor, Qualitative research, Thematic analysis, Identity

## Abstract

**Purpose:**

Eating disorders are frequently associated with fragmented self-experiences, including harsh self-criticism, shame, and withdrawal, which may be difficult to articulate using standard clinical interviews. Structured elicitation tools may support the exploration of these internal self-states in a clinically meaningful way.

**Methods:**

A total of 14 women receiving treatment for eating disorders at a specialised Italian centre participated in a structured elicitation interview using nine visual–symbolic self-part cards developed for clinical use. For each card, participants provided a numeric endorsement rating (0–10) and open-ended explanations following fixed prompts. Data were analysed using an inductive thematic approach with cross-case comparison.

**Results:**

Higher endorsement was observed for cards reflecting self-rejection, internal criticism, and concealment. Across participants’ accounts, four cross-cutting themes emerged: (1) persecutory self-criticism linked to internalised relational judgement; (2) shame-based body rejection as a core self-reference; (3) protective withdrawal associated with emotional invisibility and (4) developmental discontinuity characterised by forced maturity and unmet childhood needs.

**Conclusions:**

This brief report suggests that structured elicitation using visual–symbolic prompts can facilitate the exploration of clinically relevant self-states in people with eating disorders. Findings are preliminary but highlight the potential value of this approach for assessment and psychotherapy formulation.

**Level of evidence:**

Level V, qualitative descriptive study based on clinical interviews.

**Supplementary Information:**

The online version contains supplementary material available at 10.1007/s40519-026-01857-1.

## Introduction

Eating disorders are severe psychiatric conditions characterised by disturbances in eating behaviour, body image, and weight-related concerns, often accompanied by significant psychological distress and medical complications [NO_PRINTED_FORM]. Beyond diagnostic criteria, they are increasingly conceptualised as complex, multidimensional disorders shaped by the interaction of biological, psychological, and sociocultural factors. From a clinical perspective, the lived experience of an eating disorder frequently involves conflicting internal self-states, including harsh self-criticism, shame, and withdrawal, which may alternate in their influence on thoughts, emotions, and behaviour [1].

Empirical and clinical research has highlighted that individuals with eating disorders often report difficulties in emotion identification, regulation, and integration, alongside elevated levels of self-criticism and dissociative tendencies [2, 3]. Beyond cognitive and affective descriptions, these internal self-states may also be understood in relation to embodiment, namely the way bodily experience contributes to the construction of selfhood and personal identity. In eating disorders, body-related shame, self-rejection, and emotional disconnection are not only correlates of psychopathology, but may reflect altered ways of inhabiting, perceiving, and evaluating the body as a lived self. From this perspective, self-states such as concealment, denial, or self-criticism may be interpreted not only as intrapsychic positions, but also as embodied modes of organising subjective experience, particularly when bodily and emotional signals are difficult to integrate [4]. These features may contribute to rigid and punitive internal configurations that sustain eating disorder symptoms and interfere with recovery processes.

In clinical practice, the use of “parts” language has gained attention as a pragmatic way to explore the multiplicity of the self, particularly in individuals with complex relational histories. Within this perspective, subjective experience is understood as involving interacting self-states that may serve protective, avoidant, or self-punitive functions, rather than as a unitary and coherent self [5]. Accessing these internal self-states, however, can be challenging using standardised interviews or self-report measures, as they are often experienced implicitly and described with difficulty.

Structured elicitation tools using visual–symbolic prompts have been developed in therapeutic contexts to support the externalisation and articulation of internal self-states [6]. In the context of eating disorders, these prompts may be particularly useful in accessing embodied and affectively charged aspects of self-experience that are difficult to articulate through conventional interviews alone. Compared with unstructured qualitative interviewing, this approach provides shared symbolic anchors and a common set of prompts across participants, thereby increasing procedural consistency and cross-case comparability. At the same time, unlike standardised self-report instruments, it allows participants to elaborate the personal meaning, developmental origin, and perceived function of the elicited self-states in their own words. In this sense, the cards were used as structured elicitation devices intended to facilitate access to experiences that may remain implicit, affectively charged, or difficult to verbalise spontaneously. When adapted for research purposes, such tools may allow for a more systematic exploration of how individuals with eating disorders describe the origin, function, and perceived influence of their internal self-states, while maintaining clinical relevance.

The present exploratory qualitative study used nine visual–symbolic self-part cards within a structured elicitation interview to address the following research questions: (1) how women with eating disorders describe and organise their internal self-states when prompted by visual–symbolic cards, and (2) how these self-states are perceived to influence eating-related behaviours and emotional experience.

## Methods

### Participants and setting

Participants were recruited from the ED ward of Villa Margherita (Arcugnano–Vicenza, Italy), a specialised inpatient and day-hospital centre for the treatment of eating disorders. All participants were women aged 18 years or older and were receiving treatment for a clinically confirmed eating disorder at the time of the study.

A total of 14 women took part. The mean age was 22.79 years (SD = 4.54, 18–32), and the mean BMI at the time of admission was 18.72 kg/m^2^ (SD = 5.16). Diagnostic distribution, based on DSM-5 criteria, was: bulimia nervosa (BN; *n* = 6), anorexia nervosa restricting type (AN-R; *n* = 5), and anorexia nervosa binge–purge type (AN-BP; *n* = 3). Duration of illness ranged from 1 to 15 years (mean = 6.9 years).

Diagnosis was established by experienced clinicians through a semi-structured clinical interview based on DSM-5 criteria for eating disorders. This assessment was part of routine clinical practice at the Centre and was used to confirm eligibility for participation in the study.

The study was conducted in accordance with the Declaration of Helsinki and was approved by the Vicenza Ethics Committee as part of a longitudinal project evaluating treatment outcomes. Participation was voluntary, and all participants provided written informed consent prior to inclusion. Given the clinical vulnerability of the sample, interviews were conducted by trained clinicians, and participants were reminded they could stop or skip questions at any time without consequence to their treatment. All data were anonymised prior to analysis to ensure confidentiality.

### Materials: metaphorical inner world self-part cards

The interview employed a set of nine visual–symbolic self-part cards originally developed for therapeutic use in clinical practice [7]. Each card depicts a distinct internal self-state through an image accompanied by a brief descriptive statement. In this study, the cards were used exclusively as structured elicitation prompts, not as analytic categories. All nine cards were presented to each participant. Participants were free to indicate that a card did not resonate with their experience. For transparency, a brief description of each card is provided in Table 1. The materials are proprietary and covered by licence; access to the cards can be requested from the author (NS).

**Table 1:** Metaphorical self-part cards used in the structured interview

Card name	Brief description
The Rejected Self	Embodies body image distortion and prevents acceptance of the body as it is; linked to fear of returning to a previously rejected physical state and to shame about perceived flaws
The Piranha	Represents a deeply internalised critic that undermines self-esteem and filters reality through negativity, often echoing external critical voices
The Chubby Self	Maintains a persistent self-concept of being “chubby,” regardless of current weight; resistant to change and associated with the belief that approval must be earned through appearance
The Hidden Self	Protects by remaining invisible and avoiding exposure due to past experiences of harm; linked to secrecy and mistrust
The Denying Self	Refuses to acknowledge emotions, pain, or illness, functioning through unawareness and resistance to change. Believes that problems are best solved by denying them
The Impostor Self	Fears being “found out” as inadequate or fake; driven by the belief that others would withdraw love or approval if the true self were revealed
The Unseen Child	Feels ignored or invisible; seeks attention through emotional shifts or behaviours in order to be noticed and loved
The Girl Who Never Was	Avoids emotions and relationships, relying on control. Represents premature maturity and self-reliance developed to meet external demands before being ready, often involving early role reversal and unmet needs
The Girl Who Couldn’t Never Grow Up	Represents a self-part formed from unmet childhood needs and developmental arrest, with difficulty assuming adult roles and fear of autonomy. Often closely linked to The Girl Who Never Was

Original card content developed for clinical use within therapeutic practice. Descriptions adapted for research transparency

#### Interview procedure

Interviews were conducted individually in a quiet room within the treatment facility at the beginning of the inpatient treatment protocol and prior to the initiation of psychotherapeutic sessions. Cards were presented one at a time in a fixed order. Cards were treated as visual–symbolic elicitation prompts and were not analysed as linguistic metaphors. For each card, participants were asked to respond to the same set of open-ended prompts, focusing on: (a) whether and how the depicted self-state related to their experience; (b) its perceived origin; (c) situations in which it was most influential, including eating-related behaviours and interpersonal contexts; and (d) its perceived protective or harmful functions. Participants were also asked to identify which cards felt most representative of themselves and to explain their choices. For each card, participants provided an endorsement rating from 0 (not at all representative) to 10 (extremely representative). Ratings ≥ 7 were used descriptively to indicate strong identification. Interviews lasted between 45 and 75 min, were audio-recorded with consent, and transcribed verbatim. This structured format was intended to preserve narrative openness while ensuring that all participants were invited to reflect on comparable experiential domains, including origin, function, eating-related influence, and interpersonal impact. The combination of fixed prompts and open-ended elaboration was chosen to support both clinical depth and cross-case analytic comparison.

#### Qualitative analysis

Data were analysed using reflexive thematic analysis [8]. Analysis proceeded through repeated familiarisation with the transcripts, initial coding of meaning units relevant to participants’ descriptions of internal self-states, and iterative development of themes capturing recurrent patterns across cases. Themes were generated inductively across the dataset and were not equivalent to the card labels. Although the cards provided predefined symbolic prompts, they were not treated as analytic categories and did not determine the thematic structure a priori. We acknowledge, however, that the elicitation procedure likely shaped the narrative field by making some domains of experience more readily available for reflection. For this reason, themes were developed across transcripts and across cards, with particular attention to recurrent experiential functions that extended beyond the wording of individual card labels. Analytic decisions were discussed within the research team to refine theme boundaries and ensure coherence. Two researchers with expertise in eating disorder phenomenology independently coded the transcripts and resolved discrepancies through discussion. Reflexive notes and a structured codebook were maintained to document analytic decisions. Initial open coding generated a broad set of descriptive codes capturing content, emotional tone, and perceived function. Through iterative comparison and team discussion, overlapping codes were merged and conceptually related codes were grouped into higher-order themes based on shared experiential functions. Codes that were infrequent or insufficiently supported across cases were discarded during refinement. Themes were developed inductively across the dataset rather than per card and subsequently organised into broader functional groupings to reflect clinically meaningful patterns of self-organisation. Given the exploratory aims and the clinical nature of the sample, findings are intended to be descriptive and hypothesis-generating rather than generalisable. Interview prompts are reported in Supplementary Table S1. The coding framework and theme development are reported in Supplementary Table S2; cross-case theme coverage is reported in Supplementary Table S3.

## Results

All nine cards were presented to each participant. Five cards were most frequently identified as strongly representative of participants’ experience (endorsement ≥ 7): the Rejected Self (*n* = 9), the Piranha (*n* = 8), the Chubby Self (*n* = 7), the Impostor Self (*n* = 6), and the Hidden Self (*n* = 6). These cards primarily reflected experiences of self-criticism, shame, and withdrawal, suggesting that such self-states were central in participants’ accounts (Table 1).

Following this descriptive overview of card endorsement, thematic analysis was conducted across all transcripts to identify recurrent patterns of experience. Themes were derived inductively from participants’ accounts and were not based directly on card labels. Rather, they reflected recurrent experiential dimensions that emerged across multiple cards and participants within the structured elicitation context. The resulting cross-cutting themes reflect shared experiential dimensions emerging across multiple cards and participants.

### Cross-cutting themes

#### Persecutory self-criticism

A pervasive theme across accounts was the presence of harsh, punitive self-states characterised by conditional self-worth, perfectionism, and relentless self-monitoring. Participants described these self-states as automatically activated, particularly in situations involving food or body exposure. One participant noted: “At the table, the thought is already there: you can’t eat” (20-year-old, AN), highlighting how self-criticism directly shaped eating behaviour.

Beyond meals, similar self-states influenced broader self-evaluation and interpersonal functioning: “If I don’t meet certain standards, I feel completely worthless” (25-year-old, BN). These accounts suggest that persecutory self-criticism operated not only as a symptom-related process but also as a dominant internal mode organising self-experience.

#### Shame-based body rejection

Participants frequently described a persistent sense of bodily defectiveness and rejection that remained stable despite changes in weight or treatment progress. This self-state was often rooted in earlier experiences of comparison or criticism and continued to shape present self-perception. As one participant explained: “I still carry the things they used to call me” (28-year-old, BN), indicating the enduring influence of past evaluations.

Shame-based body rejection was closely linked to oscillations between restriction and binge eating, as well as to compulsive body monitoring. In several accounts, the body itself became the primary site of self-judgement, reinforcing a rigid and negatively valence self-reference.

#### Protective withdrawal and emotional avoidance

Another recurring theme concerned self-states oriented towards protection through withdrawal, invisibility, or emotional denial. Participants described these self-states as strategies aimed at reducing exposure to perceived relational or emotional threat. One participant stated: “If I don’t show myself, I can’t get hurt” (19-year-old, AN), summarising the defensive logic underlying withdrawal.

While experienced as protective, these self-states were also associated with isolation, secrecy, and difficulty accessing emotional needs. In some cases, withdrawal was mirrored in eating-related behaviours, such as secretive binge eating or avoidance of shared meals: “I used to hide my binges because I was ashamed” (24-year-old, BN).

#### Developmental discontinuity

Several accounts reflected self-states associated with disrupted developmental trajectories, characterised either by premature responsibility or by difficulty assuming adult roles. Participants described feeling forced to mature early, often at the expense of emotional expression or dependency needs: “I had to be the adult in the house” (20-year-old, AN).

Conversely, other self-states were associated with fear of autonomy and uncertainty in adult functioning: “Growing up means leaving, and that’s terrifying” (21-year-old, AN). These experiences suggest a tension between early hyperfunctionality and developmental arrest, contributing to instability in self-organisation.

#### Clinical organisation of self-states

From a descriptive clinical perspective, participants’ self-states could be broadly organised into those oriented towards internal punishment and control and those oriented towards protection through withdrawal or avoidance. Cards reflecting punitive and withdrawal-oriented functions were most frequently endorsed as central to participants’ experience, suggesting that these modes play a prominent role in the subjective organisation of eating disorder psychopathology in this clinical sample.

## Discussion

This brief qualitative study explored how women with eating disorders describe internal self-states using a structured elicitation procedure based on visual–symbolic prompts. Across participants’ accounts, self-states oriented towards self-criticism, shame, and withdrawal were consistently identified as central to subjective experience. Cards reflecting punitive and avoidant functions were more frequently endorsed than those referring to developmental themes, suggesting that self-states directly involved in current distress and behavioural regulation may be more accessible and salient in this clinical context.

The cross-cutting themes identified in the analysis—persecutory self-criticism, shame-based body rejection, protective withdrawal, and developmental discontinuity—are consistent with the clinical descriptions of eating disorder psychopathology emphasising rigid self-evaluation, emotional avoidance, and difficulties in self-regulation [9, 10]. These findings may also be understood from an embodiment perspective. Themes such as body rejection, concealment, and shame-based self-evaluation suggest that the body was not merely the object of dissatisfaction, but a central site through which participants organised self-experience, relational anticipation, and emotional regulation. In this sense, the elicited self-states appear linked not only to cognitive self-evaluation, but also to altered embodied self-reference, consistent with recent work highlighting the role of embodiment and interoceptive processes in eating disorders and related conditions [4]. Rather than representing discrete or static entities, these self-states appeared to function as dominant modes of experience shaping both eating-related behaviours and interpersonal functioning. The frequent association between critical self-states and restrictive or controlling behaviours supports models that conceptualise eating disorder symptoms as strategies for managing internal distress rather than solely as weight- or shape-driven concerns [1].

From a clinical perspective, the process of selecting and reflecting on self-part cards appeared to facilitate access to aspects of experience that participants described as difficult to articulate spontaneously. From a methodological perspective, the card-based elicitation procedure appeared to offer a useful intermediate format between open qualitative interviewing and more standardised assessment. The shared visual prompts and fixed questions created a common narrative scaffold across participants, while preserving the possibility for individual elaboration and unanticipated meanings to emerge. Rather than functioning as explanatory models, the cards served as structured elicitation devices that supported access to internal experiences, which participants described as difficult to articulate spontaneously. This may help explain why cards linked to self-criticism and withdrawal were more readily endorsed, whereas those referring to developmental vulnerability were less frequently selected and may require greater therapeutic safety to be explored.

Overall, the findings support the usefulness of structured elicitation methods for examining self-organisation in eating disorders. By focusing on participants’ own descriptions of internal states and their perceived functions, this approach offers a clinically grounded complement to standardised assessments. Rather than proposing a new theoretical model, this brief report provides preliminary evidence that visual–symbolic prompts can support the exploration of self-states relevant to symptom maintenance and emotional regulation, generating hypotheses for future, larger-scale studies.

### Limitations

This study involved a small, single-centre sample of women receiving treatment for eating disorders, which limits generalisability. As with all qualitative research, results reflect participants’ accounts within a specific interview context. An additional limitation concerns the use of predefined visual–symbolic prompts. Although the themes were developed inductively and were not derived from the card labels, the cards may have increased the salience of specific domains of experience and thus influenced what participants chose to elaborate during the interview. Therefore, the findings should be considered descriptive and hypothesis-generating.

## Conclusion

This brief report suggests that structured elicitation using visual–symbolic self-part prompts can facilitate the exploration of clinically relevant self-states in women with eating disorders. Self-states centred on self-criticism and withdrawal were particularly salient, highlighting their role in subjective distress and behavioural regulation. While preliminary, these findings underscore the potential value of this approach for assessment and psychotherapy formulation and warrant further investigation in larger and more diverse samples.

### Strength and limits

A key strength of this brief report is the use of a structured elicitation procedure to explore subjective self-states in individuals with eating disorders, combining fixed prompts with open-ended responses to enhance cross-case comparability while preserving clinical depth. The integration of descriptive endorsement ratings with inductive thematic analysis supports analytic transparency in a small clinical sample.

Several limitations should be acknowledged. The sample size was limited and consisted exclusively of women receiving treatment in a specialised centre, which restricts generalisability. In addition, the cross-sectional design does not allow conclusions regarding changes in self-states over the course of treatment or recovery.

### What is already known on this subject

Previous research has shown that eating disorders are frequently associated with harsh self-criticism, shame, and difficulties in self-organisation. Qualitative and clinical models have described the presence of conflicting internal self-states and defensive patterns such as withdrawal and control, highlighting their relevance for understanding eating disorder psychopathology and its persistence over time.

### What this study adds

This brief report provides preliminary empirical evidence that structured elicitation using visual–symbolic self-part prompts can facilitate the exploration of clinically relevant self-states in people with eating disorders. Across participants’ accounts, recurring themes of persecutory self-criticism, shame-based body rejection, protective withdrawal, and developmental discontinuity were identified. These findings suggest that this approach may offer a clinically useful complement to standard assessment methods by supporting reflection on internal self-states involved in distress and behavioural regulation, with potential implications for psychotherapy formulation.

## Supplementary Information

Below is the link to the electronic supplementary material.

Supplementary file 1.

## Data Availability

Data supporting the findings of this study are available from the corresponding author, upon reasonable request.
