# Supplementary material for: Understanding inner self-states in eating disorders: preliminary findings from a structured elicitation study
Source: Eat Weight Disord. 2026 Apr 15;31(1):36. doi: 10.1007/s40519-026-01857-1 (PMC13091891; doi:10.1007/s40519-026-01857-1)
Supplement: Supplementary file 1 [file 40519_2026_1857_MOESM1_ESM.docx]

Supplementary Table S1

| **Interview phase** | **Prompt** | **Analytic focus** | **Example follow-up** |
| --- | --- | --- | --- |
| Card resonance | “Does this card represent a part of you? In what way?” | Identification of self-state | “Can you tell me when you first recognised this part?” |
| Origin | “When do you think this part developed?” | Developmental context | “What was happening in your life at that time?” |
| Function | “What does this part try to do for you?” | Protective / punitive function | “How does it help you? How does it harm you?” |
| Eating behaviour | “How does this part influence eating or body-related behaviours?” | Symptom linkage | “What happens at meals when this part is active?” |
| Interpersonal impact | “How does this part affect relationships?” | Relational positioning | “Does it bring you closer or make you withdraw?” |
| Salience | “Which parts feel most representative of you?” | Subjective centrality | “Why these and not the others?” |

Supplementary Table S2. Coding framework and development of themes

| **Initial code** | **Code definition** | **Illustrative quote** | **Final theme** |
| --- | --- | --- | --- |
| Automatic self-criticism | Internal judgments that arise without conscious control, particularly in relation to food or body | “At the table, the thought is already there: you can’t eat.” (20y, AN) | Persecutory self-criticism |
| Conditional self-worth | Self-evaluation dependent on meeting rigid standards | “If I don’t meet certain standards, I feel completely worthless.” (25y, BN) | Persecutory self-criticism |
| Persistent body defectiveness | Enduring sense of bodily flaw resistant to change | “I still carry the things they used to call me.” (28y, BN) | Shame-based body rejection |
| Avoidance through invisibility | Withdrawal to reduce emotional or relational exposure | “If I don’t show myself, I can’t get hurt.” (19y, AN) | Protective withdrawal |
| Emotional numbing | Reduction of emotional awareness to prevent distress | “It’s easier not to feel anything.” (28y, BN) | Protective withdrawal |
| Premature responsibility | Feeling forced to assume adult roles early | “I had to be the adult in the house.” (20y, AN) | Developmental discontinuity |
| Fear of autonomy | Anxiety related to independence and adult roles | “Growing up means leaving, and that’s terrifying.” (21y, AN) | Developmental discontinuity |

Supplementary Table S3. Cross-case coverage of core themes (N = 14)

| **Cross-cutting theme** | **Core codes (summary)** | **Participants showing theme, n/N (%)** |
| --- | --- | --- |
| Theme 1 — Persecutory inner critic and internalised judgment | Punitive self-talk; internalised critical voices; shame; conditional self-worth | 12/14 (85.7%) |
| Theme 2 — Shame-based body rejection | Persistent body defectiveness; rejection sensitivity; body-related shame; eating-related regulation | 14/14 (100%) |
| Theme 3 — Protective withdrawal and invisibility | Emotional withdrawal; avoidance; concealment; mistrust | 10/14 (71.4%) |
| Theme 4 — Developmental discontinuity and “unlived self” | Premature responsibility; developmental arrest; tension between autonomy and dependence | 11/14 (78.6%) |

**Note.** Theme presence indicates that a participant articulated content consistent with the theme at least once during the interview. Percentages are descriptive and do not imply quantitative prevalence. This matrix is provided to document cross-case thematic coverage and support analytic transparency in an exploratory qualitative study.
